# Development and Characterization of Three Novel FGFR Inhibitor Resistant Cervical Cancer Cell Lines to Help Drive Cervical Cancer Research

**DOI:** 10.3390/ijms26051799

**Published:** 2025-02-20

**Authors:** Nauf Bou Antoun, Hiba-Tun-Noor Afshan Mahmood, Anthony J. Walker, Helmout Modjtahedi, Richard P. Grose, Athina-Myrto Chioni

**Affiliations:** 1 School of Life Sciences Pharmacy and Chemistry, Department of Biomolecular Sciences, Kingston University London, Kingston-upon-Thames KT1 2EE, UK; k1741364@kingston.ac.uk (N.B.A.); t.walker@kingston.ac.uk (A.J.W.); h.modjtahedi@kingston.ac.uk (H.M.); 2 Centre for Tumour Biology, Barts Cancer Institute, Queen Mary University of London, London EC1M 6BQ, UK; r.p.grose@qmul.ac.uk

**Keywords:** drug resistance, cervical cancer, FGF(R), HeLa, CaSki, SiHa, cervical cancer treatment

## Abstract

Primary or acquired resistance to therapeutic agents is a major obstacle in the treatment of cancer patients. Cervical cancer is the fourth leading cause of cancer deaths among women worldwide and, despite major advances in cancer screening and treatments, many patients with advanced stage cervical cancer have a high recurrence rate within two years of standard treatment, with drug resistance being a major contributing factor. The development of cancer cell lines with acquired resistance to therapeutic agents can facilitate the comprehensive investigation of resistance mechanisms, which cannot be easily performed in clinical trials. This study aimed to create three novel and robust cervical cancer cell lines (HeLa, CaSki, and SiHa) with acquired resistance to a fibroblast growth factor receptor (FGFR) tyrosine kinase inhibitor (PD173074). All three drug-resistant (DR) cell lines overexpressed FGFR1, FGFR2, FGF2, FGF4, and FGF7 proteins that were also localized to the nucleus. In addition, the DR cells had a significantly more aggressive phenotype (more migratory and proliferative, less apoptotic) compared to the parental cell lines. These novel DR cervical cancer cells are a critical tool for understanding the molecular mechanisms underpinning drug resistance and for the identification of potential cervical cancer biomarkers. Moreover, the availability of such DR cell lines may facilitate the development of more effective therapeutic strategies using FGFR inhibitors in combination with other agents that target pathways responsible for acquired resistance to FGFR inhibitors.

## 1. Introduction

Cancer is a complex cellular disease characterized by its heterogeneity, which is impacted by multiple genetic, epigenetic, internal (microenvironment), and external environmental factors. These characteristics can make it challenging to effectively target cancer therapeutically, especially in its late stages. Cervical cancer, in particular, is largely preventable through effective primary (i.e., HPV vaccination) and secondary (i.e., Papanicolaou screening and HPV PCR testing) prevention and is curable when diagnosed early. Despite this, cervical cancer remains the fourth most common cancer among women globally; in 2022, ~350,000 women worldwide died from the disease [1,2]. The primary risk factor for cervical cancer is persistent infection with high-risk HPV types (e.g., HPV-16 and the slightly less common, but still significant, HPV-18) [3]. Although there is optimism about the potential elimination of cervical cancer through vaccination and screening, the lack of universal global access to prophylactic vaccines, the evolution of HPV subtypes, and the acquisition of multi-drug resistance complicates efforts to control and eradicate the disease [4].

Resistance to current cancer therapies is a significant challenge that can reduce treatment efficacy and often result in poor outcomes in patients with cervical cancer [5]. For example, in cervical cancer, its resistance to cisplatin is well documented [6,7,8]. Cancer cells utilize diverse mechanisms to become resistant to interventions [9]. For example, the tumor microenvironment (TME), which is comprised of many cellular and acellular components, can provide a protective niche for cancer cells [10]. Enhanced angiogenesis due to hypoxia within the TME can alter cellular metabolism [11]. Furthermore, acquired mutations in a protein target can hamper its interaction with a drug. For example, in cervical cancer, the *TP53* gene is often altered, leading to chemotherapy resistance and poor progression-free survival [12,13,14]. In addition, epigenetic modifications (i.e., DNA methylation, histone acetylation, and non-coding RNA regulation) can alter the expression of genes involved in drug resistance (e.g., silencing of tumor suppressor genes) [15,16,17,18]. The overexpression of drug efflux pumps (e.g., P-glycoprotein) can reduce intracellular drug concentrations and the cytotoxic effects of cancer drugs by actively transporting them out of the cell [19,20]. Chemotherapies that target proteins involved in homologous recombination repair can increase the activity of DNA repair mechanisms and, therefore, allow cancer cells to survive certain chemotherapies and radiation that utilize DNA damage as their mode of action [21]. Cancer cells can also develop mechanisms to avoid apoptosis induced by chemotherapeutic treatment, such as the upregulation of anti-apoptotic (e.g., B-cell lymphoma 2 (Bcl-2)) and the downregulation of pro-apoptotic (e.g., Bcl-2-associated protein x (Bax)) proteins [22]. Ultimately, it is important to improve our understanding of the mechanisms used by cancer cells to become treatment-resistant to help drive the development of alternative cancer therapies, including therapies for cervical cancer. This can be achieved by employing combination therapies, targeting specific resistance mechanisms, and personalizing treatment approaches [23].

One example of targeted cancer therapy is the use of tyrosine kinase (TK) inhibitors (TKIs), a class of small-molecule compounds that target the activity of TKs. These enzymes are important components that drive downstream signaling cascades that orchestrate cellular proliferation, migration, survival, and differentiation [24,25]. These TKIs are approved to be used, and have shown promise, as treatment in a number of haematological and solid malignancies in an attempt to target pathways driven by receptor TKs (RTKs), such as vascular endothelial growth factor receptors (VEGFRs), platelet-derived growth factor receptor (PDGFR), FGFR, stem cell factor receptor (KIT), rearranged during transfection (RET) (e.g., each targeted by Regorafenib), mesenchymal epithelial transition (MET) (Gabozantinib), and epidermal growth factor receptor (EGFR) (e.g., erlotinib and gefitinib) [26]. As with other cancer treatments, cells can also develop resistance mechanisms to TKIs [26,27]. FGFRs are members of the RTK family and are essential for the attainment of normal cellular homeostasis, particularly during’ embryogenesis and development [28,29]. Given the involvement of FGFRs in wide-ranging processes such as cell proliferation, differentiation, organogenesis, angiogenesis, neurogenesis, bone development, wound healing, and tissue repair [30,31], it is unsurprising that this RTK also has an essential role in the evolution of cancer (e.g., breast, ovarian, endometrial, lung, and cervical cancer) [28,32] and that many TKIs used in clinical trials target the FGFR axis (e.g., erdafitinib, pemigatinib, infigratinib, derazantinib, futibatinib and AZD4547 (AZD)) [33,34,35].

Previously, we have demonstrated the importance of FGFR signaling in cervical cancer by activating the FGFR axis with FGF ligand in the presence or absence of an FGFR inhibitor (PD173074) and monitoring the functional consequences [36]. The FGF-induced activation of the FGFR axis was accompanied by increased cancer cell proliferation and migration, and reduced apoptosis. These effects were reversed or abolished upon co-treatment with PD173074 (PD) [36]. This study expands on our previous work [36], addressing the critical issue of drug resistance in cervical cancer by developing a suite of robust cell line models (SiHa, CaSki, and HeLa) that are resistant to an FGFR inhibitor to facilitate future mechanistic studies that dissect drug resistance pathways.

## 2. Results

### 2.1. Establishing PD173074-Resistant Cervical Cancer Cell Lines

**Figure 1:**
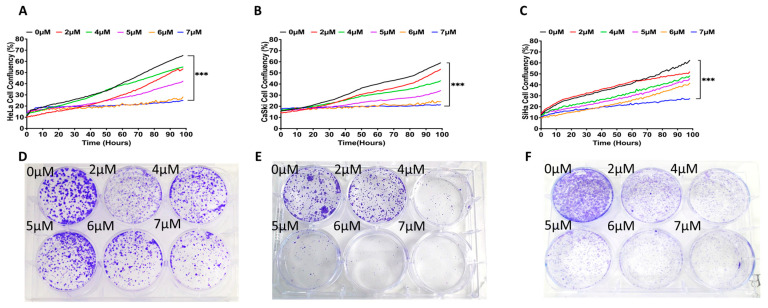

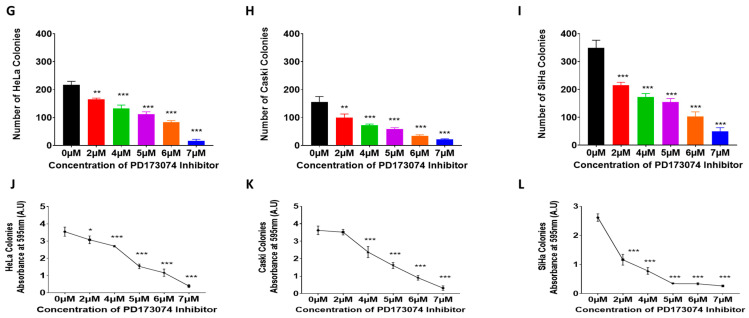
Effect of the FGFR1 inhibitor, PD173074, on CCCL growth. The CCCLs HeLa (**A**,**D**,**G**,**J**), CaSki (**B**,**E**,**H**,**K**), and SiHa (**C**,**F**,**I**,**L**) were plated at low confluence and (**A**–**C**) with their confluency monitored over time in the presence of increasing concentrations of PD173074 (2 μM–7 μM) or DMSO control (0 μM). (**D**–**F**) A visual representation (at day 12) of the six-well culture plate in which CCCL colonies were formed and stained with crystal violet. (**G**–**I**) Graphical representation of the number of colonies present for each of the different concentrations of PD173074. The colonies were enumerated by eye, each colony contains >50 cells. Graphs (**J**–**L**) represent the colonies shown; the crystal violet was dissolved and the absorbance was measured at 595 nm for each treatment. Graphical data represent the mean (±SEM) of three independent experiments and differences between mean values were analyzed with one-way ANOVA followed by Tukey’s post-hoc multiple comparison test; * *p* ≤ 0.05, ** *p* ≤ 0.01, *** *p* ≤ 0.001, when compared with control (DMSO) values.

The three human cervical cancer cell lines (HCCCLs) employed here, HeLa, CaSki, and SiHa, were initially treated with increasing concentrations (from 2 μM to 7 μM) of the small-molecule FGFR inhibitor PD173074 to establish an effective dose for long-term treatment to support the development of drug resistance. Incucyte cell proliferation and crystal violet colony formation assays revealed significantly reduced cell growth (Figure 1A–C; *p* < 0.001) and colony formation (Figure 1D–L; *p* < 0.01 above 2 μM) in all three cell lines and with all PD173074 concentrations used. After four days of incubation, 6 and 7 μM of PD173074 almost completely abrogated the HeLa and CaSki cell proliferation (Figure 1A,B). In the case of the SiHa HCCCL (Figure 1C), although both concentrations dramatically reduced the cell growth by ~75–~90%, 7 μM of PD173074 had the most prominent effect. With all of the results taken together, the amount of 5 μM of PD173074 was selected for further study given that it was the concentration with the most impactful, but non-lethal, effect on the cells over time.

### 2.2. Profiling FGF(R) Protein Expression, Secretion, and Activation in PD173074-Resistant Cell Lines

**Figure 2:**
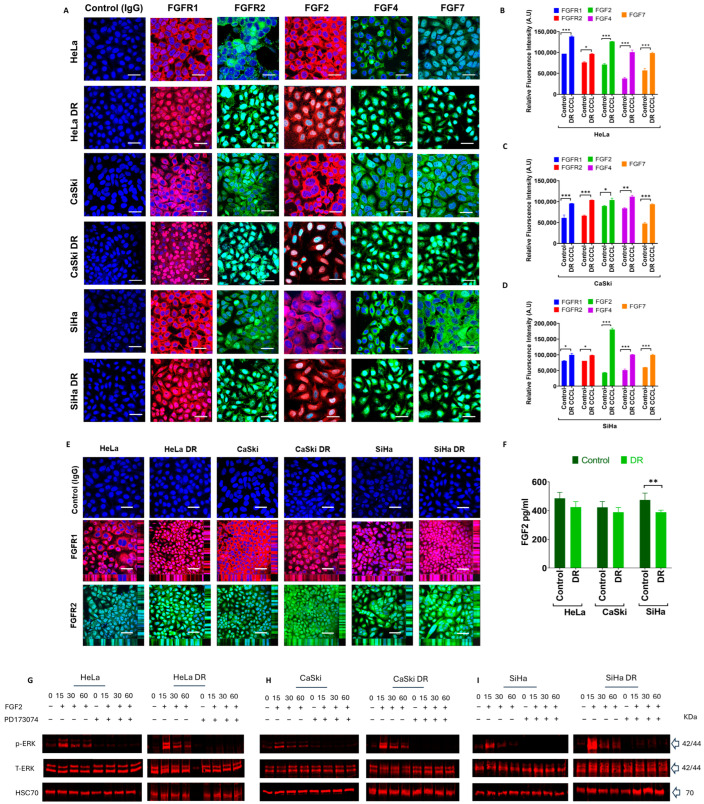
Mapping FGF receptor and FGF ligand protein expression in PD173074-resistant CCCLs. (**A**) FGFR and FGF protein expression in parental and drug-resistant (DR) HeLa, CaSki, and SiHa CCCLs revealed by immunocytochemistry. FGFR1 (red), FGFR2 (green), FGF2 (red), FGF4, and FGF7 (both green) expression were all visibly greater in all three DR cell lines; FGFR1, FGFR2, FGF2, FGF4, and FGF7 ligands were predominately localized in the nucleus, cytoplasm, and plasma membrane. Negative control cells were incubated with immunoglobulin G (IgG) from the same species as the primary antibody (rabbit for FGFR1, FGFR2, FGF4, and FGF7; mouse for FGF2). Nuclei were stained with DAPI (blue); scale bar, 50 μm. (**B**–**D**) Relative fluorescence intensities of FGFs/FGFRs were quantified using ImageJ (v1.54p, Wayne Rasband, National Institute of Health (NIH), Bethesda, MD, USA) in parental and DR CCCLs. (**E**) Confocal z-stacks confirmed the nuclear localization of FGFR1 (red) and FGFR2 (green) in parental and DR HeLa, CaSki, and SiHa CCCLs, and it was at apparently higher levels in all three DR cell lines. Negative control cells were treated as in ‘A’; scale bar, 50 μm. (**F**) FGF2 secretion was determined using ELISA after 48 h culture; SiHa parental cells secreted more FGF2 compared to their corresponding DR cell line. (**G**–**I**) Erk phosphorylation (p-ERK; activation) after FGF2 stimulation in parental (**G**) HeLa, (**H**) CaSki, (**I**) SiHa versus their corresponding DR CCCLs. The CCCLs were stimulated for 15, 30, and 60 min, ±2 μM PD173074 with FGF2 ligand, and displayed ERK phosphorylation between 15 and 60 min in both parental and DR cell lines. However, with PD173074, the increase in phosphorylation was abolished in both cell lines. The data represent the mean (± SEM) of three independent experiments. Differences between means (compared to control) were analyzed with (**B**–**D**) two-way ANOVA followed by Dunnett’s post-hoc multiple comparison test and (**F**) one-way ANOVA followed by Tukey’s post hoc test; * *p* ≤ 0.05, ** *p* ≤ 0.01, *** *p* ≤ 0.001.

Next, all three CCCLs were treated for nine months with 5 μM of PD173074, similar to the duration employed to develop gefitinib-resistant cell lines in non-small cell lung cancer (NSCLC) [37]. Once the drug-resistant (DR) cells were established, they were screened for FGFR1, FGFR2, FGF2, FGF4, and FGF7 proteins, the main receptors/ligands expressed in normal CCCLs, as determined previously [36]. Interestingly, acquired resistance to the FGFR TKI was accompanied by an increased expression of FGFR1 and FGFR2 proteins in all three DR CCCLs (Figure 2A–D). In addition, there was an apparent increased nuclear expression of these receptors in all three DR CCCLs (Figure 2A,E; confocal z-stacks can be viewed on the borders of each image in Figure 2E and Figure S3). In terms of the protein expression of the FGFR ligands, all three DR CCCLS had increased staining for FGF2, FGF7, and FGF4, which was particularly evident in the nucleus (Figure 2A).

The drug-resistant cells were also compared with their parental counterparts to investigate whether their FGF secretion was altered (Figure 2F). The cells were grown in serum-free conditions for 48 h prior to collection of the media for ELISA analysis. The cell proliferation assays did not reveal any significant differences in cell number between the parental and DR CCCLs over the 48 h (Figure S1A), possibly due to the serum-free conditions making them less metabolically active. All three CCCLs secreted FGF2 but not FGF4 or FGF7 into the medium (Figure S1B,C). Overall, in the parental and DR CCCLs, the FGF2 ligand concentrations reached between 370 and 490 pg/mL after 48 h of culture (Figure 2F). For the HeLa and CaSki cells, there was no significant difference in FGF2 secretion between the parental and DR cell lines (Figure 2F). However, the FGF2 secretion was significantly lower (*p* < 0.01) in the DR SiHa cells compared with their parental cell line, with ~388 pg/mL and ~472 pg/mL being secreted, respectively (Figure 2F). Such findings suggest that parental and DR CCCLs might activate FGFR through an autocrine loop.

The extracellular signal-regulated kinase (ERK) pathway is known to be activated via the FGFR axis [36], so we hypothesized that this pathway could be mechanistically affected in the new DR cells. However, the acquired resistance of the CCCLs to the FGFR TKI did not appear to alter their activation of ERK signalling. Despite the cells being resistant to PD173074, the FGF2 induced ERK phosphorylation (p-ERK; commensurate with ERK activation) in all CCCLs and was abolished with treatment with 2 μM PD173074 in the parental cells, as expected, but also in their equivalent DR cell lines (Figure 2G–I).

### 2.3. Morphological and Functional Characteristics of PD173074-Resistant Cervical Cancer Cell Lines

**Figure 3:**
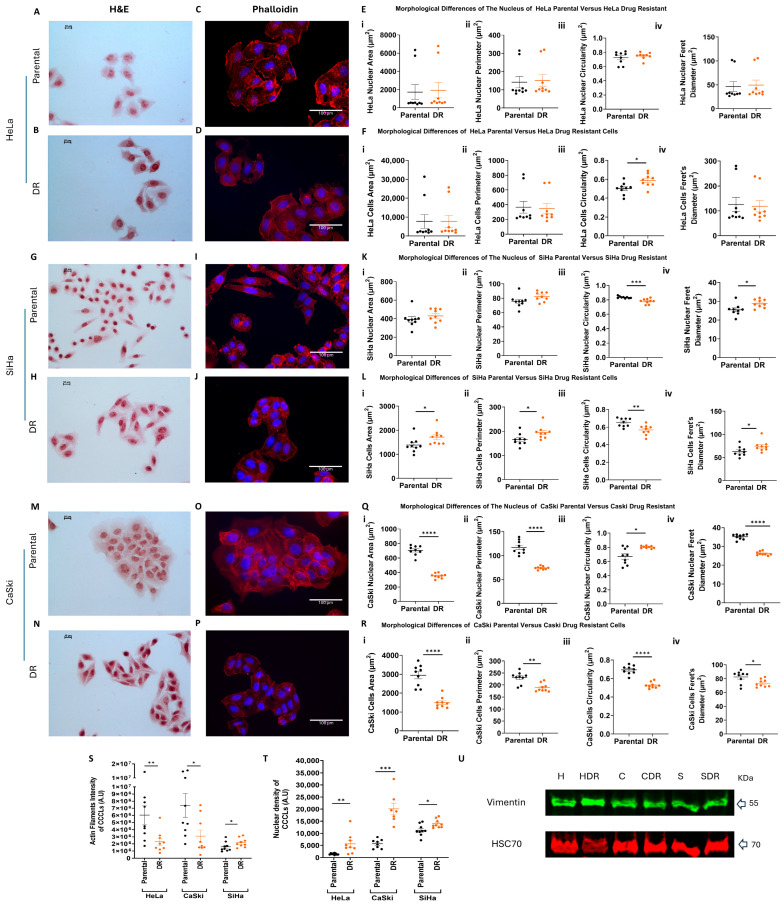
Cytological differences between parental and PD173074-resistant CCCLs. Images of H&E stained (**A**) HeLa parental and (**B**) drug-resistant (DR) cells, (**G**) SiHa parental and (**H**) DR cells, (**M**) CaSki parental and (**N**) DR cells; nuclei are stained purple, cytoplasm is stained pink; scale bar 10 µm. Phalloidin staining of (**C**) HeLa parental and (**D**) DR cells, (**I**) SiHa parental and (**J**) DR cells, (**O**) CaSki parental and (**P**) DR cells; nuclei (DAPI) are stained blue, cytoskeletal F-actin (rhodamine phalloidin) is stained red; scale bar 100 µm. Pictures taken on (**A**,**B**,**G**,**H**,**M**,**N**) Nikon DS-Fi2 microscope (×40 objective) and (**C**,**D**,**I**,**J**,**O**,**P**) Evos digital inverted microscope (×40 objective). Analysis of morphological differences between (**E**) HeLa parental and DR cells, (**K**) SiHa parental and DR cells, (**Q**) CaSki parental and DR cells at the nuclear level ((**i**) nuclear area, (**ii**) nuclear perimeter, (**iii**) nuclear circularity, (**iv**) nuclear Feret diameter) and (**F**) HeLa parental and DR cells, (**L**) SiHa parental and DR cells, (**R**) CaSki parental and DR cells at the cellular level ((**i**) cellular area, (**ii**) cellular perimeter, (**iii**) cellular circularity, (**iv**) cellular Feret diameter) after DAPI and phalloidin staining. Expression of (**S**) actin filaments and (**U**) vimentin in all parental and DR CCCLs (*n* = 3). (**T**) Nuclear densitometry of H&E staining in parental and DR CCCLs. H: HeLa, HDR: HeLa DR, C: CaSki, CDR: CaSki DR, S: SiHa, SDR: SiHa DR. The data represent the mean (±SEM) of three independent experiments. Differences between means were analyzed by *t*-test followed by Mann–Whitney test; * *p* ≤ 0.05, ** *p* ≤ 0.01, *** *p* ≤ 0.001, **** *p* ≤ 0.0001.

Overall, the gross morphologies of the DR cells were similar to those of their parental counterparts (Figure 3). The analysis of various nuclear and cellular parameters revealed that the HeLa DR cells displayed a similar nuclear and cellular area, perimeter circularity, and Feret diameter (Figure 3A–F), although the cells did become significantly more circular (Figure 3(Fiii)). In contrast, the DR SiHa and CaSki cells became less circular (Figure 3(Liii,Riii), respectively). However, the SiHa DR cells also exhibited reduced circularity of the nucleus (Figure 3(Kiii)), while the CaSki DR cells showed the opposite, with their nuclei being more circular than those of their parental cells (Figure 3(Qiii)). In addition, the CaSki DR cells exhibited a significant reduction in both their nuclear and cellular area and perimeter (Figure 3(Q,Ri,Rii)), whereas the SiHa DR cells had an increased cell area and perimeter (Figure 3(Li,Lii)) without any significant change in their nuclear dimensions, apart from their circularity being slightly decreased (Figure 3(Ki–Kiii)). The nuclear and cellular Feret diameter remained unchanged in the Hela DR cells (Figure 3(Eiii,Fiii)) but was increased in the SiHa DR (Figure 3(Kiii,Liii)) and decreased in the CaSki (Figure 3(Qiii,Riv)) cells. 

Interestingly, the DR HeLa and CaSki cells displayed reduced staining of filamentous actin, while the actin staining was greater in the SiHa DR cells compared to their parental counterparts, supporting that DR cells possess a modified actin network (Figure 3S). The H&E staining demonstrated that all three DR CCCLs (Figure 3B,H,N,T) had darker nuclear staining compared to their parental counterparts (Figure 3A,G,M,T), consistent with the more cancerous phenotype of actively dividing cells due to increased chromatin density and nucleic acid content (e.g., active transcription of RNA) whilst preparing for cell division. Vimentin, which is a hallmark of epithelial to mesenchymal transition (EMT) and is associated with a more metastatic cell behaviour, was expressed in all three parental and DR CCCLs at comparable levels (Figure 3U).

**Figure 4:**
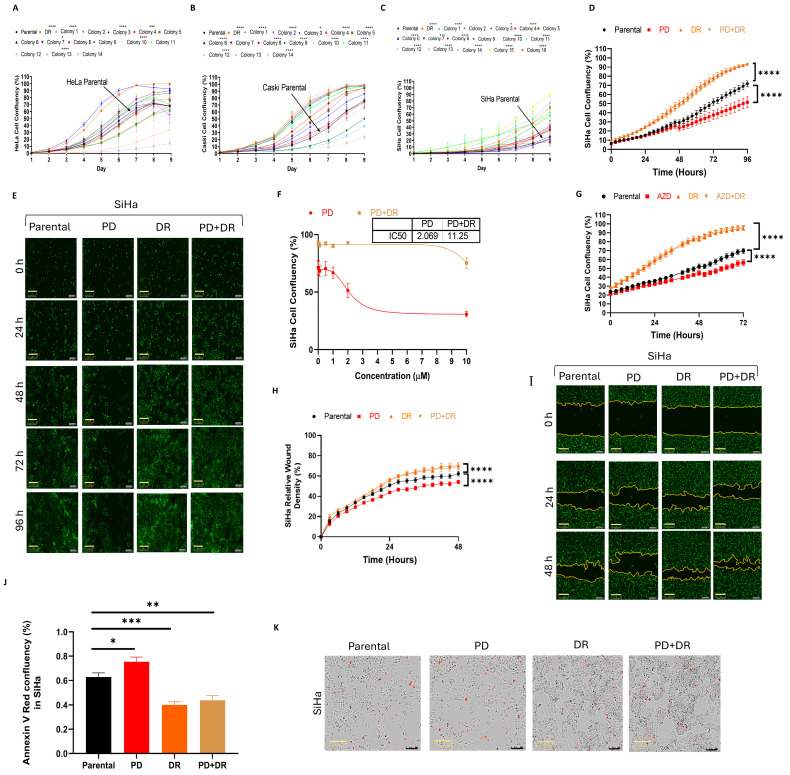
PD173074-resistant SiHa cells proliferate and migrate faster, are less apoptotic, and have an IC50 greater than that of the parental cell line. Parental versus drug-resistant (DR) colony cell proliferation assays showing, when compared to the parental heterogeneous cell line: (**A**) colony 3 of HeLa DR cell line proliferating faster and colony 13 proliferating slower; (**B**) colony 2 of CaSki DR cell line proliferating faster and colony 14 proliferating slower; (**C**) colony 15 of SiHa DR cell line proliferating faster and colony 14 proliferating slower. (**D**) H2B-GFP transfected SiHa parental and DR cell lines treated with 2 μM PD173074 (PD) or DMSO (control). (**E**) Selection of images at ×10 magnification captured by IncuCyte Zoom system (2018A, Essen Bioscience, Ann Arbor, Michigan, USA) showing the proliferation rate of cells exposed to ±2 μM PD173074 or DMSO between SiHa parental and DR cells. Scale bar, 300 μm. (**F**) IC50 curves of SiHa parental versus SiHa DR calculated after the cells were treated with PD173074 for 96 h, showing cell confluency (%) of SiHa parental versus DR cells treated at increasing concentrations of PD173074 (0, 0.01, 0.1, 0.5, 1, 2, and 10 μM). (**G**) SiHa parental and DR cell lines were treated with 2 μM AZD4547 (AZD) or DMSO. (**H**,**I**) A 700–800 μm-wide wound was created by a wound maker in SiHa parental and DR cell lines and media ±2 μM PD173074 was added. (**I**) Selection of images at ×10 magnification from IncuCyte Zoom (2018A, Essen Bioscience, Ann Arbor, Michigan, USA) showing the difference in wound closure rate between SiHa parental and DR cells. Scale bar, 300 μm. (**J**,**K**) SiHa parental cells ±2 μM PD173074 with IncuCyte^®^ Annexin V Red Reagent (4641; Essen BioScience, Ann Arbor, MI, USA) show a higher apoptotic rate than the DR lines after 24 h. (**J**) The cell red object confluence (%) was measured using the IncuCyte software (2018A, Essen Bioscience, Ann Arbor, Michigan, USA). (**K**) Selection of images at ×10 magnification from Incucyte Zoom system showing the difference in apoptotic rate between SiHa parental and DR cells. The red dots show apoptotic cells. Scale bar, 300 μm. The data represent the mean of three independent experiments (± SEM). Differences between means were analyzed with (**D**,**G**,**H**) two-way ANOVA or (**J**) one-way ANOVA, followed by Tukey’s post-hoc test; * *p* ≤ 0.05, ** *p* ≤ 0.01, *** *p* ≤ 0.001, **** *p* ≤ 0.0001.

Several clones were selected from each of the three DR cervical cancer cell populations, and cell proliferation assays confirmed their heterogeneity, with some clones exhibiting greater proliferation rates compared to others (Figure 4A–C). The proliferation was also assessed between the mixed DR populations and their equivalent parental CCCLs (HeLa, CaSki, and SiHa) in the presence and absence of two FGFR inhibitors, PD173074 and AZD4547 (Figure 4D–G, Figures S2, S4A–D and S5A–D). For simplicity and because the data were consistent with all three cell lines, the SiHa cell line data are presented here, and the data from the other two cell lines are provided within the Supplementary Materials (Figures S4 and S5).

The SiHa DR cells treated with either PD173074 (Figure 4D) or AZD4547 (Figure 4G), or neither, possessed higher proliferation rates compared to their parental equivalents that were treated or not treated with the same inhibitors (Figure 4D,E,G). The IC50 value for PD173074 increased ~5.5 fold from 2.069 μM (parental cells) to 11.25 μM (DR cells) (Figure 4F). These functional results indicate that the DR cell lines have become resistant to further treatment with the FGFR inhibitors, despite the fact that these same treatments could attenuate the activation of p-ERK when the cells were stimulated with FGF2 (Figure 2G–I). Because the mixed population of DR cells responded well in functional studies and because they were a more representative cervical cancer model due to their heterogenicity, further experiments were performed using these cells and not a selected clone with higher proliferation rates (Figure 4A–C). 

Next, scratch wound healing assays were performed to determine the migratory behaviour/proliferation of each cell line. Concomitant with the effects observed in the cell proliferation assays, the DR cells closed the wound significantly faster than the parental cells, and treatment with the FGFR TKI PD173074 did not have any effect on the wound closure of the DR CCCLs (Figure 4H,I). Furthermore, compared to the parental cells, the DR CCCLs were also less susceptible to PD173074- induced apoptosis (Figure 4J,K).

### 2.4. No Mutations Exist in the FGFR1 TK Domain of the Three PD173074-Resistant Cervical Cancer Cell Lines

**Figure 5:**
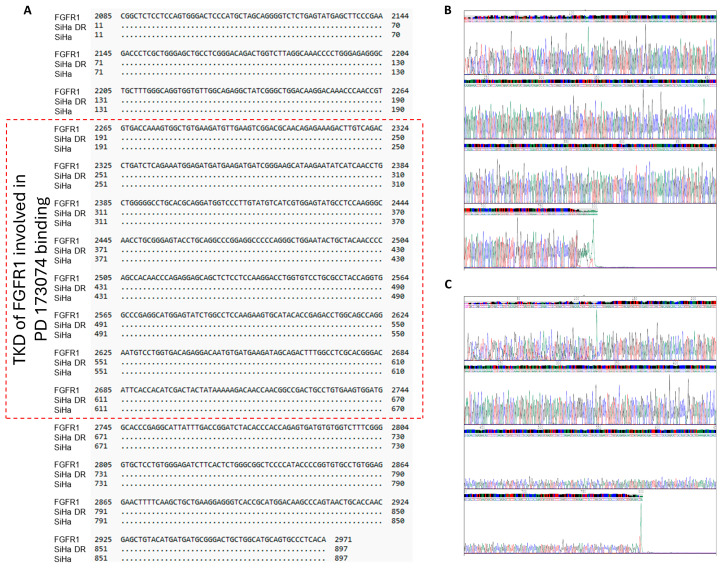
PD173074-resistant cell lines do not possess mutations in the FGFR1 TK domain (TKD). (**A**) Alignment of the TKD of FGFR1 (NM_023110.3) to that of SiHa parental and DR cells. PD173074 binds to FGFR1 within the area delimited by the red dashed lines in the TKD. Chromatographs of (**B**) SiHa parental and (**C**) SiHa DR cells using the second set of forward primers. Similar data were seen in the other CCCLs.

Because the DR cells, when compared with their parental counterparts, displayed clear functional differences, both with and without FGFR inhibitors, samples were prepared and sequenced by Sanger sequencing to determine whether they had acquired any mutation(s) in the FGFR1 TK domain (TKD). Such mutation(s) could affect drug binding and/or FGFR phosphorylation/activation, although the cells responded to FGF ligand and blockade with PD173074 at biochemical levels (Figure 2G–I). Sanger sequencing revealed no mutations in the TKD of FGFR1 in all three of the DR CCCLs (Figure 5, Figures S6 and S7).

## 3. Discussion

Resistance to anti-cancer therapy, including targeted therapy or traditional cytotoxic chemotherapies, in advanced or recurring cervical cancer is a major problem that can lead to poor outcomes. To our knowledge, this study represents the first time HCCCLs that are resistant to an FGFR TKI have been generated and characterized. The drug-resistant cell lines provide a robust tool to identify and further elucidate potential genes, proteins, and signaling pathways involved in cancer drug resistance.

All three generated DR cell lines were a heterogeneous population, as expected, mimicking the true nature of cancer. The blockade of FGFR signaling has an inhibitory effect on cell proliferation, as described previously [36]. The characterized DR cells had increased levels of receptor (FGFR1 and FGFR2) proteins as well as ligand (FGF2, FGF4, and FGF7) proteins, compared with their parental cells. Interestingly, these proteins were also detected at higher levels in the nucleus, consistent with previous findings on cervical, breast, and pancreatic cancer suggesting that the total FGF(R) and nuclear localization are associated with more metastatic behaviour [36,38,39,40]. This agrees with findings that nuclear FGFR1 in oestrogen receptor-positive tumors was positively correlated with anti-oestrogen therapy (fulvestrant) resistance [41]. However, the CCCLs only secreted FGF2, and not the other FGF ligands. This suggests that, whilst the other ligands were retained intracellularly, FGF2 might be a critical driver for promoting cancer progression by affecting cancer cells via autocrine or, in tumor microenvironments, via paracrine signaling similar to that described in pancreatic cancer [38]. 

The present study has confirmed previous findings [36] that the activation of the FGFR axis via FGF2 leads to ERK activation and that pre-treatment with PD173074, an FGFR small-molecule inhibitor, blocked this response. However, the DR cells also responded to the inhibitor treatment, which blocked ERK activation, suggesting that the inhibitor can still bind to the FGFR TKD and that the activation of other pathways (i.e., phosphoinositol-3-kinase/protein kinase B (PI3K/AKT)) might play a compensatory role. The DR cells might have developed partial resistance, with other pathways being upregulated to compensate for and bypass the FGFR activation [9,42]. This could involve the activation of other RTKs or downstream effectors that drive proliferation, migration, and cell survival. It is also plausible that the heterogeneity of our cell population masks some effects, with some cells retaining sensitivity to PD173074 and others not and developing resistance mechanisms. This might explain the observed inhibition of the FGFR–ERK axis after PD173074 treatment in the overall population, while a more aggressive phenotype is still maintained in terms of the functional studies. Moreover, even though PD173074 inhibited FGFR–ERK signaling, the DR cells might have developed mechanisms to rapidly reactivate the pathway or compensate through other means. For example, in FGFR inhibitor-resistant lung cancer cells, NRAS amplification and DUSP6 deletion resulted in mitogen-activated protein kinase (MAPK, i.e., ERK) reactivation [43]. All these aspects are currently under investigation in our laboratory. 

Mutations in the TKD of FGFR1 frequently modify the activation loop or the adenosine triphosphate (ATP)-binding pocket of the kinase, causing structural alterations that obstruct drug binding. For example, mutations in the FGFR1 kinase domain, including N546K and V561M, confer resistance to the FGFR TKIs ponatinib and erdafitinib [44,45]. These substitutions either directly alter the drug’s affinity for the receptors’ ATP-binding site or stabilize FGFR’s active conformation, which makes it more difficult for inhibitors to bind [46]. Furthermore, FGFR1 TKD mutations can cause conformational changes that enhance downstream signaling and further promote cancer cell survival. For example, certain mutations result in elevated protein kinase activity that is independent of ligand binding [47]. Additionally, these mutations can cause drug resistance by activating other signaling pathways including PI3K/AKT or ERK, compensating for the attenuated FGFR activation [48,49]. In the current study, Sanger sequencing confirmed that the DR cells did not have any mutations in their TKD that would affect PD173074 drug binding and subsequent FGFR activation. For example, valine (Val)559 and Val561, which determine selectivity, and alanine (Ala)564, glutamic acid (Glu)562, lysine (Lys)514, aspartic acid (Asp)641, Lys514, Glu531, methionine (Met)535, isoleucine (Ile)545, Val559, Val561, Ala640, and phenylalanine (Phe)642, which are important for the binding of PD 173074 to the ATP-binding cleft [50,51,52,53,54], were not mutated in any of the three DR CCCLs.

Mutations outside the TKD can also drive drug resistance. For example, S249C mutation in the extracellular domain of FGFR3, in association with other mutations (e.g., K650E and K650M in TKD), can have strong transforming activities associated with drug resistance [45]. FGFR2 extracellular domain mutations can alter the function of the receptor and potentially influence drug resistance in patients with FGFR2-fusion-positive immunocytochemistry [45,55]. Mutations in the transmembrane domain (e.g., FGFR3 Y373C mutation) and in the juxtamembrane domain might also influence drug responses [45,55,56]. Further characterization of the genetic alterations supporting the generated DR cells may highlight mechanisms of the observed change in phenotypes (e.g., growth and migration).

Despite the lack of mutations in the TKD and their persistent sensitivity to PD173074 in the context of p-ERK blockade, the DR cells were more proliferative and migratory and less apoptotic compared to their equivalent parental cells. Importantly, treatment with two FGFR inhibitors (PD173074 and AZD4547) did not reduce their proliferative and migratory capacities; furthermore, PD173074 did not increase apoptosis in the DR cells. These findings support the conclusion that all three DR cell lines were insensitive to both of the two FGFR inhibitors at the functional level. In addition, the PD173074 IC50 in the DR cells was ~5.5-fold higher than that of the parental cells. Although, according to some reports, resistance is indicated by an increase in IC50 of 10 times or more [57], a two- to three-fold increase is considered the lower threshold for resistance [58]. The magnitude of the IC50 change observed in this study is frequently reported as a strong signal of resistance in the context of resistance to targeted treatments or chemotherapeutics. For instance, studies on TKIs in resistant cancer cell lines have demonstrated that, depending on the mutations and resistance mechanisms involved, IC50 values might increase from 5 to over 50 times [59,60,61].

Because cancer cells are typically more migratory and invasive, resisting cell death, and, as in this study, even more so when they become DR, they typically undergo some morphological changes. For example, they may lose their typical epithelial characteristics and become more fibroblast-like, displaying a more spindle-shaped morphology, a loss of epithelial polarity, nuclear positioning, and a more elongated shape, as well as sometimes having cell protrusions (e.g., filopodia or lamellipodia). This change is referred to as the EMT (e.g., [62]). In addition, due to changes in their growth rate and cell metabolism, DR cells might appear larger in size and possess an increased nucleus size due to increased transcriptional activity and chromatic remodeling [63,64]. Although there were no consistent changes across all three DR cells, nuclear enlargement was observed in the SiHa DR cells; H&E hyperchromasia was also observed in all three DR cells, an indication that they are more proliferative.

Whilst EMT is usually associated with drug resistance, this relationship is complex and can vary based on the cancer and drug type. It is plausible that the CCCLs already possessed some EMT features before they became DR, a notion supported by the similar expression levels of vimentin found in all three parental and DR CCCLs. In addition, drug resistance in cancer cells can cause substantial cytoskeletal alterations that may affect the shape of the cell without producing the elongated, mesenchymal phenotype that is commonly linked to EMT. These alterations may entail the rearrangement of microtubules and actin filaments, resulting in non-mesenchymal alterations in cell shape [65,66,67,68].

Another possibility is that the DR cells generated here possess an intermediate epithelial/mesenchymal phenotype or a partial or intermediate EMT, a plasticity that has been previously reported to be associated with drug resistance [69,70,71,72]. In this state, certain mesenchymal characteristics, such enhanced motility or resistance to apoptosis, are acquired by the cells while they maintain certain epithelial characteristics, including cell–cell attachment. Cells in this ‘hybrid’ state exhibit a different morphology from their parental counterparts despite not entirely elongating or becoming spindle-shaped [73]. Drug resistance has been associated with this sort of partial EMT, which can produce a phenotype that is halfway between epithelial and mesenchymal. Interestingly, according to recent data, cancer cells that have undergone partial or hybrid EMT naturally have a high degree of flexibility and the ability to initiate metastases, but cancer cells that have undergone full EMT have less potential for metastatic spread. It has also been suggested that EMT-dependent mechanisms control the dissemination of tumor cells as clusters by collective migration [74,75,76,77].

Drug-resistant cancer cells frequently change shape to adapt to their treatment environment and microenvironment. This adaptation, as opposed to the dispersed, migratory phenotype observed in cells undergoing EMT, may cause cells to become more compact or take a form that promotes survival under stress, which might explain why the Hela DR cells became more circular. Rather than a whole shift to a mesenchymal state, these adaptations could result from modifications in cell–matrix and cell–cell adhesion characteristics [78,79,80]. For instance, a morphology that is more rounded or irregular due to resistance to targeted therapies (especially those that block pathways involved in cell survival and proliferation) may result from a compromise between maintaining certain epithelial characteristics and adjusting to the stress imposed by a drug [81,82,83,84]. The cell shape can also be affected by modifications in the ways that cells interact with the matrix that surrounds them. DR cells may exhibit a morphology that is neither completely dispersed nor clearly mesenchymal due to changes in their attachment to the extracellular matrix (ECM) or in their expression of integrins and other cell adhesion molecules and tight junctions [85,86]. When the cells adjust to new ECM circumstances or modified mechanical signals from the microenvironment, these modifications may produce a morphology that is more rounded or compact. Therefore, in future investigations, it would be meaningful to interrogate more EMT markers in these cell lines (beyond vimentin, e.g., E-cadherin, Snail, and Slug) and also study cell–cell and ECM interactions in DR and parental cervical cancer cells in 3D organotypic models that are more realistic than 2D culture.

In summary, our findings demonstrate that all three DR CCCLs exhibited a significant upregulation of FGFR1, FGFR2 FGF2, FGF4, and FGF7 protein expression. Notably, this increased expression was particularly pronounced in the nucleus. In addition, the DR CCCLs possess a more metastatic signature, and they are resistant to the FGFR inhibitor PD173074 in terms of proliferation, migration, and apoptosis, despite the lack of an FGFR TKD mutation. Therefore, these three CCCLs can be used as a reliable model to study signaling mechanisms involved in drug resistance. It would be of particular interest to investigate genes, proteins, and signaling mechanisms that might differ between all three parental and DR cells in the future, with a view to applying the resulting knowledge to more clinically based studies. This can be done by identifying: (i) predictive biomarkers for drug resistance and cells’ response to treatment; (ii) molecular targets for combination therapies; (iii) new drug development/novel compounds against resistant cells; (iv) mutations or alterations associated with acquired resistance; (v) assays to monitor treatment responses by tracking resistance-associated markers in real-time; and (vi) molecular profiles of patients to be able to determine an informed protocol or treatment.

## 4. Materials and Methods

### 4.1. Cell Culture

Three HCCCLs, CaSki, SiHa, and HeLa, were purchased from American Type Culture Collection (ATCC, Manassas, VA, USA) and the three DR HCCCLs were generated using a potent ATP-competitive FGFR inhibitor, PD173074 (P2499; Sigma-Aldrich, St. Louis, MO, USA). The HCCCLs were cultured in Dulbecco’s modified Eagle’s medium (DMEM) (D5796; Sigma-Aldrich, St. Louis, MO, USA) supplemented with 10% fetal bovine serum (FBS) (F9665; Sigma-Aldrich, St. Louis, MO, USA) and 200 µg·mL^−1^ penicillin–streptomycin (P4333; Sigma-Aldrich, St. Louis, MO, USA). Cells were grown under sterile conditions in a humidified incubator at 37 °C/5% CO_2_ and passaged when at 80% confluence.

### 4.2. Establishment of PD173074-Resistant Cell Lines

Parental HCCCLs (HeLa, SiHa, and CaSki) were cultured at 37 °C in medium containing 10% FBS, with a confluency of 1 × 10^3^ cells per well in 6-well plates (3516; Corning costar treated plates, Corning, NY, USA). Following a 24 h period, the medium was replenished, and the cells were treated with several concentrations of PD173074 (0, 2, 4, 5, 6, and 7 μM) or DMSO as negative control and incubated at 37 °C. The medium/drug was changed every two days. After 10 days incubation, the medium was discarded and the cells were rinsed with phosphate buffered saline (PBS), then fixed with 4% formaldehyde and stained with 0.5% crystal violet. Each well was photographed, and the cells were manually counted. The ultimate concentration of PD173074 selected to create the DR cell lines was 5 μM, and these cell lines were consistently cultured at the same drug concentration until they developed resistance (after nine months) and for the duration of the project to maintain resistance.

More specifically, the DR cells were maintained by supplementing their media with 5 μM PD173074, which was replenished every two days for two passages, then the dose of PD173074 was gradually increased to 5.5 μM and 6 μM for the subsequent passage. The DR cells were cultured without the drug for a week before performing each experiment.

### 4.3. PD173074-Resistant Colony Formation

Heterogeneous DR CCCLs were plated in 6-well tissue culture plates (3516; Corning costar treated plates, Corning, NY, USA) at varying confluences, ranging from 100 to 3000 cells per well. The plates were then left to incubate for 10 days in media containing 10% FBS, or until each colony had approximately 20 cells. Under a microscope, each colony was examined and carefully removed to generate a new culture. they were then each transferred into a well of a 96-well plate (3596; Corning costar treated plates, Corning, NY, USA) and incubated until they reached 70–80% confluency. Thereafter, cultures were transferred to 24-well tissue culture plates (3524; Corning costar treated plates, Corning, NY, USA), then transferred to 6-well plates, and then lastly to T 75 flasks (156499; Nunc Easy Flask 75cm Nunclon Delta Surface, Thermo Scientific, Manassas, VA, USA). Each colony was treated with 5 μM PD173074 throughout these procedures and was subsequently used in proliferation assays.

### 4.4. Functional Studies

Cells were stably transfected with the histone subunit (H2B)-GFP (11680; Addgene, Watertown, MA, USA) construct before they were used for the functional studies, following the steps previously described [87]. Briefly, the H2B-GFP construct was used to fluorescently label cells. HEK293T cells were co-transfected with 5 μg of the lentiviral transfer plasmid, 3.25 µg of pCMVR8.2 (12263; Addgene, Watertown, MA, USA), 1.7 μg of pMD2.G (12259; Addgene, Watertown, MA, USA) packaging plasmids, and FuGENE transfection reagent (Promega, Madison, WI, USA). The virus was extracted 48 h after transfection and kept at −80 °C. To transduce cell lines to stably express H2B-GFP (in 6-well plates), 1 mL of viral-containing supernatant was added to the target cells (~30% confluent), then medium was changed 24 h later and cells were assessed for expression of the construct after 48 h.

#### 4.4.1. Cell Proliferation

Cell proliferation assays were performed using an IncuCyte, ZOOM system (Essen Bioscience, Ann Arbor, MI, USA). A total of 5 × 10^3^ cells were cultured in medium containing 10% serum for 6 h in flat-bottom 96-well plates (3300; Corning costar, Corning, NY, USA) then treated with increasing concentrations of PD173074 (0, 0.01, 0.1, 0.5, 1, 2, and 10 μM) or DMSO (vehicle control) for 96 h (replenished every 48 h) and then placed in the Incucyte. Cells were also cultured for 24 h then treated with either 2 μM AZD4547 or DMSO for 72 h and placed in the Incucyte. Images were captured every 3 h while the plates were incubated for 96 h. A measure of culture confluence over time was obtained by quantifying the proliferation rate using quantitative kinetic processing parameters from time-lapse picture acquisition; the graphs were plotted from the time the drugs were added to the cultures. IC50 values were calculated using Graphpad Prism 9 (version 9.5.1, Dr. Harvey Motulsky, San Diego, Clifornia, USA).

Colony cell proliferation assays were performed using parental cells, DR cells, and their colonies by plating them in 96-well plates at 1 × 10^3^ confluency and culturing for 10 days in media containing 10% serum in the IncuCyte ZOOM system (2018A, Essen Bioscience, Ann Arbor, Michigan, USA), as described previously [36].

#### 4.4.2. Lateral Migration

Lateral migration assays were performed by seeding cells to ~100% confluency in reduced serum (5%) DMEM media to minimize proliferation. The monolayer was wounded using a 96-well wound maker (Essen Bioscience) for IncuCyte ZOOM (2018A, Essen Bioscience, Ann Arbor, Michigan, USA). Following wounding, the medium was withdrawn, and detached cells were removed from the wells using a PBS wash. After adding fresh medium, cells were treated for 48 h with either 2 μM PD173074 or DMSO (vehicle control). To investigate the impact of treatments on wound closure, the IncuCyte was programmed to take pictures every 3 h over a 48 h period. Cell migration was quantified in real time using ‘relative wound density %’, as described previously [36].

#### 4.4.3. Apoptosis

Apoptosis assays were performed by seeding 5 × 10^3^ cells in 96-well plates (3300; Corning costar, Corning, NY, USA). The IncuCyte Annexin V Red Reagent (4641; Essen BioScience, Ann Arbor, MI, USA) was added to the cells at a final dilution of 1:200 (as per the manufacturer’s instructions) once the cells had attained 30–50% confluency. The plate was then placed in IncuCyte ZOOM system (2018A, Essen Bioscience, Ann Arbor, Michigan, USA) and the scan interval set to every 2 h for 24 h using “red” and “phase contrast” channels. The apoptotic cells were quantified in real time using the “red object confluency (%)”.

### 4.5. Western Blot Analysis

FGF and inhibitor treatments were performed as described previously [36]. Briefly, recombinant human FGF2, FGF4, and FGF7 protein (R&D Systems, Minneapolis, MN, USA) stock solutions (0.1 µg·µL^−1^) were made in PBS and stored for up to three months at −20 °C. Prior to treatment, 2 × 10^5^ cells were plated in 6-well plates (3516; Corning costar treated plates, Corning, NY, USA) and serum-starved overnight at 70–80% confluency. For FGF treatments, cells were cultured for 15, 30, or 60 min in 100 ng·mL^−1^ FGF2, FGF4, or FGF7 ligands in the presence of 300 ng·mL^−1^ heparin. In parallel experiments, before being exposed to the ligand, cells were pre-treated for 1 h with either DMSO (0.01%) as vehicle control or 2 µM PD173074. The cells were prepared for western blotting after treatment.

An equal number of cells for each treatment was lysed using Bolt LDS sample buffer (4×) (Invitrogen, Carlsbad, CA, USA) mixed with 50 mM DTT and diluted to 2× with deionized water. Equal volumes of protein samples, containing 2 × 10^5^ cells, were loaded in each lane and separated by electrophoresis on hand-cast 10% Tris gels. Proteins were transferred onto nitrocellulose membranes, blocked with 5% bovine serum albumin (BSA), and incubated overnight at 4 °C with primary antibodies: phospho-p44/42 MAPK (T202/Y204), p44/42MAPK, (9101 and 4695, respectively; Cell Signalling Technology, Danvers, CA, USA), vimentin (RV202) (sc-32322, Santa Cruz Biotechnology, Dallas, TX, USA), or HSC70 (PA5-24624; Thermo-Scientific, Manassas, VA, USA) each diluted 1:1000 in 5% BSA/PBS. The membranes were then incubated for 1 h at room temperature with the secondary antibodies IRDye^®^ 680LT Donkey (926-68023; LI-COR, Lincoln, Nebraska, USA) and IRDye^®^ 800CW Donkey (926-32212; LI-COR, Lincoln, Nebraska, USA), diluted 1:10,000. PBS containing 0.1% Tween 20 (PBST) was used to wash the membranes between antibodies three times for 5 min each at room temperature. The Odyssey CLx infrared imaging system (LICOR-Biosciences) was used to visualize bands on membranes using the IMAGE STUDIO software (version 6.0, LI-COR Biosciences, Lincoln, Nebraska, USA) at 700 nm and 800 nm, which correspond to the red and green channels, respectively. The brightness and contrast were changed to obtain a suitable signal-to-noise ratio.

### 4.6. Immunocytochemistry and H&E Staining

Cells (0.5 × 10^5^) were seeded in 24-well plates (3524; Corning costar treated plates, Corning, NY, USA) that contained 13 mm-diameter glass coverslips at the base of each well. Once the cells reached 70–80% confluency they were fixed with 10% formalin for 15 min at room temperature.

For immunocytochemistry, fixed cells were permeabilized in 0.1% saponin/PBS for 10 min and blocked with 5% BSA in PBS for 45 min at room temperature. Cells were incubated for 1 h with primary antibodies (Table S1a) diluted in 5% BSA/PBS. After washing three times with PBS, cells were incubated for 1 h with secondary antibodies (Table S1b) diluted in 5% BSA/PBS 1:250, then washed three times with PBS and finally washed with water before being mounted with Prolong^®^ Diamond Antifade with DAPI (Molecular Probes (Eugene, OR, USA), Life Technologies (Carlsbad, CA, USA), Thermo Fisher Scientific (Manassas, VA, USA)). The EVOS microscope (Life Technologies, Carlsbad, CA, USA), Leica SP2 AOBS laser scanning confocal microscope, or Zeiss imaging system (Axio Observer ZEN 2.3 Systems) were used to acquire fluorescent images at ×40 magnification. IMAGE J (v1.54p, Wayne Rasband, National Institute of Health (NIH), USA) (developed by Wayne Rasband) was used to analyze the fluorescence intensity and morphological differences of the cells.

For H&E staining, fixed cells were stained in haematoxylin for 8 min, then rinsed in tap water for 5 min. Afterwards, they were stained in eosin for 10 s, then rinsed in 90% alcohol for 10 s and dehydrated in absolute alcohol for 15 s. Cells were then dehydrated and cleared in Histochoice and mounted with Histomount. A Nikon DS-Fi2 microscope was used to acquire images at ×40 magnification. 

### 4.7. ELISA

Serum-free conditions were used to cultivate 2 × 10^5^ cells per well in 24-well (3524; Corning costar treated, Corning, NY, USA) plates with PD173074 or DMSO (vehicle control) for 24 h. Employing the ab99979-FGF basic (FGF2) human ELISA kit (Abcam, Cambridge, UK), EHFGF4 human FGF4 kit, and EHFGF7 human FGF7 (KGF) ELISA kit (Thermo-Fisher Scientific, Manassas, VA, USA), the FGF2, FGF4, and FGF7 secretion into the media were determined. The manufacturer’s instructions were followed in the preparation of all reagent samples and standards.

### 4.8. Polymerase Chain Reaction (PCR) and Sanger Sequencing 

RNeasy (74134; Qiagen, Hilden, Germany) was used to extract total RNA, then the CCCLs’ cDNA was synthesized using the superscript TM IV cell direct cDNA synthesis system kit (18091050; Invitrogen, Life Technologies, Carlsbad, CA, USA) according to the manufacturer’s guidelines. PCR was carried out using MegaMix blue (2MMB-5; Microzone, Stourbridge, UK). Primer pairs (Table 1) were designed using NCBI Primer-BLAST (Primer3 version 2.5.0, National Center for Biotechnology Information (NCBI), Bethesda, Maryland, USA) for the TKD of FGFRs (Table 1). The NCBI reference sequence used to design primers was FGFR1 NM_023110.3. The annealing temperature was 62 °C.

After the PCR reaction was completed, the ladder (1 kb G571A; Promega, Madison, WI, USA) and PCR products were separated on a 1% agarose gel with Gel Red in a Tris-borate-EDTA (1x TBE) to check the quality of amplicons. The gels were visualized using a G Box imaging system (Syngene, Bangalore, India) and related Gene Tools imaging software (Image Lab 6.0.1). The PCR products were next submitted for Sanger sequencing at Genewiz (Azenta Life Sciences, Burlington, MA, USA), where the amplicon was purified and sequenced.

### 4.9. Statistical Analysis

Graphpad Prism 9 (version 9.5.1, Dr. Harvey Motulsky, San Diego, CA, USA) was used for statistical analysis. Each experiment was carried out at least three times, with separate independent biological replicates. Student’s *t*-test, one-way ANOVA, and two-way ANOVA were applied to the raw data as required, followed by Mann–Whitney and Tukey’s and Dunnett’s post-hoc multiple comparison tests, respectively.

## Figures and Tables

**Table 1:** PCR primers designed for FGFR TKDs.

Primer	Template Strand Length	Start	Stop	Product Length
TKD FGFR Forward (set 1) TAGGCAAACCCCTGGGAGA	19	9	27	802
TKD FGFR Reverse (set 1) AAGGTGGGTCTCTGTGAGGG	20	810	791	
TKD FGFR Forward (set 2) TGCATCCATGAACTCTGGGG	20	5	24	825
TKD FGFR Reverse (set 2) AGTTCCTCCACAGGCACAC	19	829	811	

## Data Availability

Data are contained within the article and Supplementary Materials.

## Supplementary Materials

The following supporting information can be downloaded at: https://www.mdpi.com/article/10.3390/ijms26051799/s1.

## Abbreviations

Ala	Alanine
Asp	Aspartic acid
ATP	Adenosine triphosphate
AZD	AZD4547
Bcl-2	B-cell lymphoma 2
Bax	Bcl-2-associated protein x
BSA	Bovine serum albumin
CCCLs	Cervical cancer cell lines
HCCCLs	Human cervical cancer cell lines
DMEM	Dulbecco’s modified Eagle’s medium
DR	Drug resistant
ECM	Extracellular matrix
EGFR	Epidermal growth factor receptor
ERK	Extracellular signal-regulated kinases
EMT	Epithelial-mesenchymal transition
ER positive	Oestrogen receptor positive
FBS	Fetal bovine serum
FGF	Fibroblast growth factor
FGFR	Fibroblast growth factor receptor
HPV	Human papillomavirus
IgG	immunoglobulin G
Ile	Isoleucine
ICC	Immunocytochemistry
Glu	Glutamic acid
KIT	Stem cell factor receptor
Lys	Lysine
MAPK	Mitogen-activated protein kinase
MET	Mesenchymal Epithelial Transition
Met	Methionine
TK	Tyrosine kinase
TKD	Tyrosine kinase domain
TKIs	Tyrosine kinase inhibitors
TME	Tumour microenvironment
PBS	Phosphate buffered saline
PBST	Phosphate buffered saline tween 20
PD	PD173074
PDGFR	Platelet-derived growth factor receptor
P-ERK	Phospho Extracellular signal-regulated kinase
PI3K/AKT	Phosphoinositol-3-kinase/protein kinase B
Phe	Phenylalanine
RET	Rearranged during transfection
RTKs	Receptors tyrosine kinase
VAL	Valine
VEGFR	Vascular endothelial growth factor receptor
