# Correction: Bou Antoun et al. Development and Characterization of Three Novel FGFR Inhibitor Resistant Cervical Cancer Cell Lines to Help Drive Cervical Cancer Research. *Int. J. Mol. Sci.* 2025, *26*, 1799

**DOI:** 10.3390/ijms27177598

**Published:** 2026-08-25

**Authors:** Nauf Bou Antoun, Hiba-Tun-Noor Afshan Mahmood, Anthony J. Walker, Helmout Modjtahedi, Richard P. Grose, Athina-Myrto Chioni

**Affiliations:** 1 School of Life Sciences Pharmacy and Chemistry, Department of Biomolecular Sciences, Kingston University London, Kingston-upon-Thames KT1 2EE, UK; k1741364@kingston.ac.uk (N.B.A.); afshan.hiba.hm@gmail.com (H.-T.-N.A.M.); t.walker@kingston.ac.uk (A.J.W.); h.modjtahedi@kingston.ac.uk (H.M.); 2 Centre for Tumour Biology, Barts Cancer Institute, Queen Mary University of London, London EC1M 6BQ, UK; r.p.grose@qmul.ac.uk

In the original publication [1], there was a mistake in Figure 2 as published.

The issue arose during the assembly of Figure 2A,E, which contains representative immunofluorescence images. The FEBS Journal publication [2] and the IJMS publication used data from related experiments but presented them in different contexts. In the FEBS Journal paper, the figure was designed to illustrate FGF(R) expression across different cervical cancer cell lines. In contrast, Figure 2A in the IJMS paper was assembled to compare FGF(R) expression between parental and FGFR inhibitor-resistant cell lines. During the preparation of this figure, several representative images were inadvertently incorporated into incorrect panels. In addition, some representative images from earlier work were unintentionally reused when assembling the IJMS figure. The corrected Figure 2 appears below.

The authors state that the scientific conclusions are unaffected. This correction was approved by the Academic Editor. The original publication has also been updated.

## Figures and Tables

**Figure 2:**
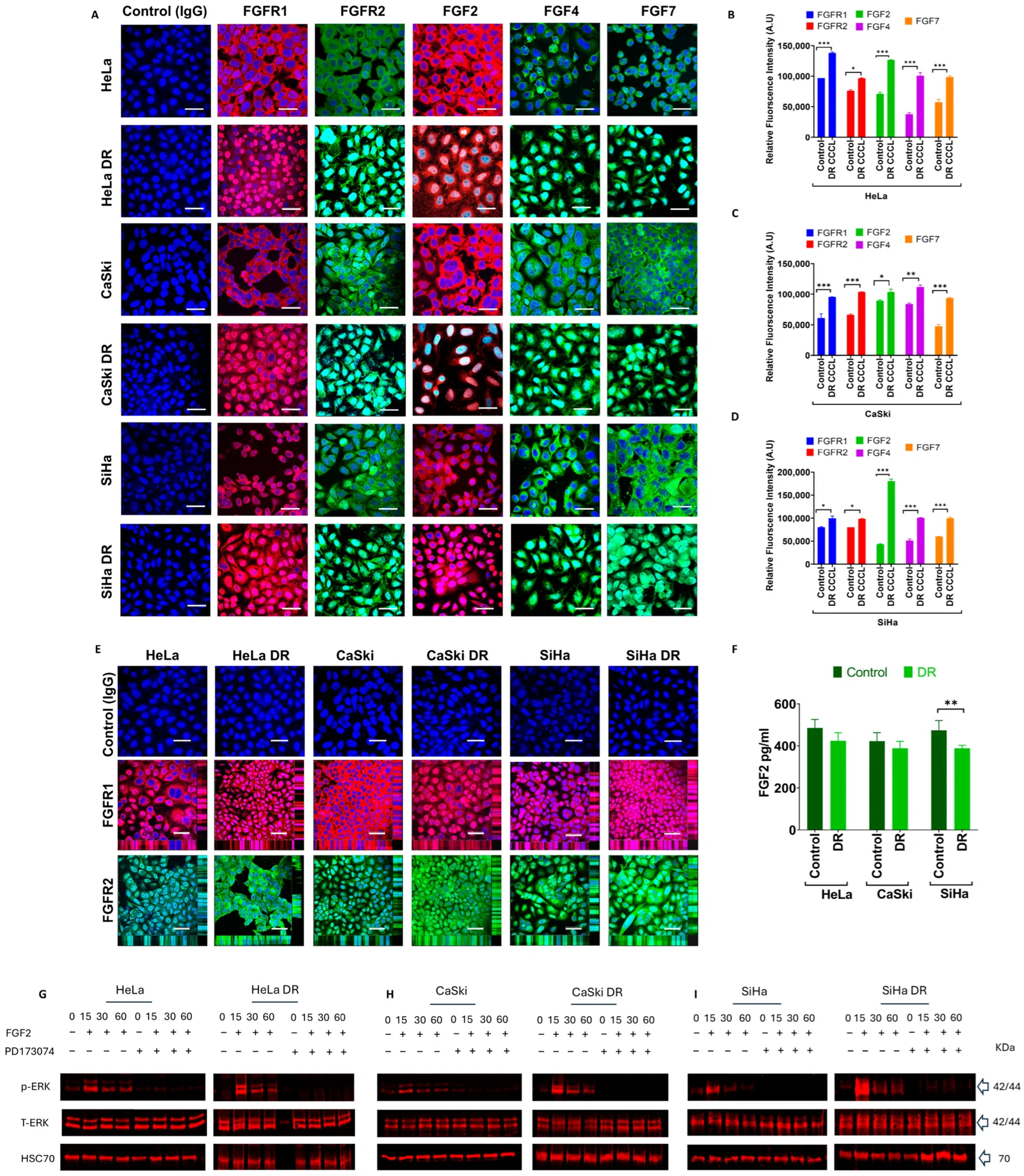
Mapping FGF receptor and FGF ligand protein expression in PD173074-resistant CCCLs. (**A**) FGFR and FGF protein expression in parental and drug-resistant (DR) HeLa, CaSki, and SiHa CCCLs revealed by immunocytochemistry. FGFR1 (red), FGFR2 (green), FGF2 (red), FGF4, and FGF7 (both green) expression were all visibly greater in all three DR cell lines; FGFR1, FGFR2, FGF2, FGF4, and FGF7 ligands were predominately localized in the nucleus, cytoplasm, and plasma membrane. Negative control cells were incubated with immunoglobulin G (IgG) from the same species as the primary antibody (rabbit for FGFR1, FGFR2, FGF4, and FGF7; mouse for FGF2). Nuclei were stained with DAPI (blue); scale bar, 50 μm. (**B**–**D**) Relative fluorescence intensities of FGFs/FGFRs were quantified using ImageJ (v1.54p, Wayne Rasband, National Institute of Health (NIH), Bethesda, MD, USA) in parental and DR CCCLs. (**E**) Confocal z-stacks confirmed the nuclear localization of FGFR1 (red) and FGFR2 (green) in parental and DR HeLa, CaSki, and SiHa CCCLs, and it was at apparently higher levels in all three DR cell lines. Negative control cells were treated as in ‘A’; scale bar, 50 μm. (**F**) FGF2 secretion was determined using ELISA after 48 h culture; SiHa parental cells secreted more FGF2 compared to their corresponding DR cell line. (**G**–**I**) Erk phosphorylation (p-ERK; activation) after FGF2 stimulation in parental (**G**) HeLa, (**H**) CaSki, (**I**) SiHa versus their corresponding DR CCCLs. The CCCLs were stimulated for 15, 30, and 60 min, ±2 μM PD173074 with FGF2 ligand, and displayed ERK phosphorylation between 15 and 60 min in both parental and DR cell lines. However, with PD173074, the increase in phosphorylation was abolished in both cell lines. The data represent the mean (± SEM) of three independent experiments. Differences between means (compared to control) were analyzed with (**B**–**D**) two-way ANOVA followed by Dunnett’s post-hoc multiple comparison test and (**F**) one-way ANOVA followed by Tukey’s post hoc test; * *p* ≤ 0.05, ** *p* ≤ 0.01, *** *p* ≤ 0.001.

## References

[1] Bou Antoun N., Afshan Mahmood H.-T.-N., Walker A.J., Modjtahedi H., Grose R.P., Chioni A.-M. (2025). Development and Characterization of Three Novel FGFR Inhibitor Resistant Cervical Cancer Cell Lines to Help Drive Cervical Cancer Research. Int. J. Mol. Sci..

[2] Mahmood H.-T.A., Tomas Bort E., Walker A.J., Grose R.P., Chioni A.-M. (2022). FGF signalling facilitates cervical cancer progression. FEBS J..

