# Supplementary material for: Development and Characterization of Three Novel FGFR Inhibitor Resistant Cervical Cancer Cell Lines to Help Drive Cervical Cancer Research
Source: Int J Mol Sci. 2025 Feb 20;26(5):1799. doi: 10.3390/ijms26051799 (PMC11898767; doi:10.3390/ijms26051799)
Supplement: Supplementary file 1 [file ijms-26-01799-s001.zip › ijms-3413592-supplementary.pdf]

Supplementary Materials:

Figure S1. FGF4 and FGF7 secretions in parental and PD173074-resistant CCCLs. (A) To verify the cell confluence 48 h before media collection, the cells were plated at a 70% confluence and cell proliferation was assessed using IncuCyte (2018A, Essen Bioscience, Ann Arbor, Michigan, USA) before ELISA. There was no significant variation in the number of cells in the proliferation assay between parental and DR CCCLs. (B) All three CCCLs' FGF4 secretion was examined using the EHFGF4 ELISA kit (Thermo-Fisher Scientific, Manassas, VA, USA) in a serum-free environment. (C) To examine FGF7 secretion in all three CCCLs, the KGF ELISA kit (Thermo-Fisher Scientific, Manassas, VA, USA) was performed in a serum-free environment. After removing the medium from the parental and DR CCCLs, ELISA was conducted; a standard curve was used to convert absorbance readings into pg/ml. The data represent the mean of three independent experiments (± SEM). Difference between means were analysed with one-way ANOVA followed by Tukey's post-hoc test.

Figure S2. Cell proliferation and apoptosis in parental and PD173074-resistant CCCLs in the presence and absence of PD173074. (A-C and D-F) In 96-well plates, parental and DR CCCLs were seeded at low confluence. They were then treated with 2 μM (A-C) PD173074, (D-F) AZD4547 or DMSO (control) for (A, D) 24 h, (B, E) 48 h and (C, F) 72 h. The cell proliferation WST-1 reagent was used to measure the cell proliferation. At 420 nm, the absorbance of each sample was measured in arbitrary units (A.U). In comparison to the control (DMSO treated cells), the parental cells (HeLa, CaSki, and SiHa) shown a significant decrease in proliferation when treated with PD173074 or AZD4547. Both untreated and treated DR CCCLs proliferate at comparable rates. The apoptotic activity of the cells (G) HeLa parental and DR, (H) CaSki parental and DR, and (I) SiHa parental and DR was assessed using the CaspGLOW Fluorescein active caspase 3 kit. To ascertain the rates of apoptosis, the cells were treated with the antibody FITC-DEVD-FMK. (G-I) Because there was higher caspase activity in the control group than in the DR group, the CCCLs exhibited more apoptotic characteristics than the DR CCCLs. DR CCCLs showed comparable apoptosis rates with and without PD173074. Relative fluorescence units (RFU) were used to measure the activity. The data represent the mean of three independent experiments (± SEM). Difference between means were analysed with (A-F) two-way ANOVA; (G-I) one-way ANOVA followed by Tukey's post-hoc test. *P≤0.05, ** P≤0.01, *** P≤0.001, ****P≤0.0001. : *P≤0.05, **P≤0.01, *** P< 0.001.

Figure S3. FGFR1 protein expression in parental and PD173074-resistant CCCLs. FGFR1 was predominately localised in the nucleus, cytoplasm and plasma membrane. Immunocytochemistry (ICC) showing uppregulation of FGFR1 expression in the nuclei and cytoplasm in all DR CCCLs compared to their parental counterparts. Nuclei is stained with DAPI (blue), FGFR1 is stained green. Pictures taken on Evos digital inverted microscope (x40 objective); scale bar, 50 μm.

Figure S4. PD173074-resistant HeLa proliferate and migrate faster, are less apoptotic and their IC50 is higher than the parental cell line. (A-B) H2B-GFP transfected HeLa parental and drug resistant (DR) cell lines treated with 2 μM of PD173074 (PD) or DMSO. (A) The graph shows the cell confluency (%) of.un/treated parental and DR cells. (B) Selection of images at x10 magnification taken by the Incucyte software (2018A, Essen Bioscience, Ann Arbor, Michigan, USA) showing the proliferation rate of cells exposed to +/- 2 μM PD173074 (PD) or DMSO between HeLa parental and DR. Scale bar, 300 μm. (C) IC50 of HeLa parental versus HeLa DR calculated after the cells have been treated with PD173074 for 96 h showing the cell confluency (%) of HeLa parental versus HeLa DR were treated at different concentrations of PD173074 (0, 0.01, 0.1, 0.5, 1, 2, and 10 μM). (D) HeLa parental and DR cell lines treated with 2 μM AZD4547 (AZD) or DMSO. (E-F) A 700-800 μm wide wound was created by a wound maker in HeLa parental and DR cell lines and medium +/- 2 μM PD173074 was added. (F) Selection of images at x10 magnification from Incucyte Zoom system (2018A, Essen Bioscience, Ann Arbor, Michigan, USA) showing the difference in wound closure rate between HeLa parental and DR. Scale bar, 300 μm. (G-H) HeLa parental cells +/- 2 μM PD173074 in the presence of IncuCyte® Annexin V Red Reagent (4641; Essen BioScience, Ann Arbor, MI, USA) show a higher apoptotic rate then the DR lines after 24 h. (G) The cell red object confluence (%) was measured using the IncuCyte ZOOM® system (2018A, Essen Bioscience, Ann Arbor, Michigan, USA). (H) Selection of images at x10 magnification taken from Incucyte Zoom system (2018A, Essen Bioscience, Ann Arbor, Michigan, USA) showing the difference in apoptotic rate between HeLa parental and DR. Scale bar, 300 μm. The red dots are the apoptotic cells. The data represent the mean of three independent experiments (± SEM). (A, D and E) Difference between means were analysed with two-way ANOVA and (G) one-way ANOVA followed by Tukey's post hoc test. * P≤ 0.05, **** P≤ 0.0001.

Figure S5. PD173074-resistant CaSki proliferate and migrate faster, are less apoptotic and their IC50 is higher than the parental cell line. (A-B) H2B-GFP transfected CaSki parental and DR cell lines treated with 2 μM of PD173074 (PD) or DMSO. (A) The graph shows the cell confluency (%) of.un/treated parental and DR cells. (B) Selection of images at x10 magnification taken by the Incucyte software (2018A, Essen Bioscience, Ann Arbor, Michigan, USA) showing the proliferation rate of cells exposed to +/- 2 μM PD173074 (PD) or DMSO between CaSki parental and DR. Scale bar, 300 μm. (C) IC50 of CaSki parental versus CaSki DR calculated after the cells have been treated with PD173074 for 96 h showing the cell confluency (%) of CaSki parental versus CaSki DR treated at different concentrations of PD173074 (0, 0.01, 0.1, 0.5, 1, 2, and 10 μM). (D) CaSki parental and DR cell lines were treated with 2 μM AZD4547 (AZD) or DMSO. (E-F) A 700-800 μm wide wound was created by a wound maker in CaSki parental and DR cell lines and medium +/- 2 μM PD173074 was added. (F) Selection of images at x10 magnification from Incucyte Zoom system (2018A, Essen Bioscience, Ann Arbor, Michigan, USA) showing the difference in wound closure rate between CaSki parental and DR. Scale bar, 300 μm. (G-H) CaSki parental cells +/- 2 μM PD173074 in the presence of IncuCyte® Annexin V Red Reagent (2018A, Essen Bioscience, Ann Arbor, Michigan, USA) show a higher apoptotic rate then the DR lines after 24 h. (G) The cell red object confluence (%) was measured using the IncuCyte ZOOM® system (2018A, Essen Bioscience, Ann Arbor, Michigan, USA). (H) Selection of images at x10 magnification from Incucyte Zoom system (4641; Essen BioScience, Ann Arbor, MI, USA) showing the difference in apoptotic rate between CaSki parental and DR. Scale bar, 300 μm. The red dots are the apoptotic cells. The data represent the mean of three independent experiments (± SEM). (A, D and E) Difference between means were analysed with two-way ANOVA and (G) one-way ANOVA followed by Tukey's post hoc test. * P≤ 0.05, ** P≤ 0.01, **** P≤ 0.0001.

Figure S6. PD173074-resistant cell lines do not possess mutations in the FGFR1 TK domain (TKD). Alignment of TKD of FGFR1 (NM_023110.3) to (A) HeLa parental and DR (D) CaSki parental and DR. PD173074 binds to FGFR1 within the area highlighted between the red lines in the TKD. Chromatographs of (B) HeLa parental, (E) CaSki parental and (C) HeLa DR, (F) CaSki DR using the second set of forward primers.

Figure S7. Chromatographs of all CCCLs forward and reverse primers. Chromatographs of forward primer (set 1) in (A) HeLa parental (E) CaSki parental, (I) SiHa parental and (B) HeLa DR, (F) CaSki DR (J) SiHa DR. Chromatographs of reverse primer (set 2) in (C) HeLa parental (G) CaSki parental, (K) SiHa parental and (D) HeLa DR, (H) CaSki DR (L) SiHa DR. Combining data from both sets of primers confirms that there are no mutation in the TKD of FGFR1 that occurred in all three DR CCCLs in response to the drug treatment. (M) Two sets of primers were used to sequence the TKD of FGFR1 (NM_023110.3), The first set of forward and reverse primers are highlighted in a green box. The second set of forward and reverse primers are highlighted in a blue box. Forward primer of set 2 is upstream of the TKD of FGFR1.

Table S1a. Primary antibodies used for immunocytochemistry assays

Primary antibodies	Host/ Isotype	Catalogue Number	Company	Working Concentration
FGFR1	Rabbit	Ab10646	Abcam	1:100
FGFR2	Rabbit	SC-122	Santa Cruz	1:100
FGF2	Mouse	05-188	Millipore	1:100
FGF4	Rabbit	Ab106355	Abcam	1:100
FGF7	Rabbit	SC7127	Santa Cruz	1:100
Rhodamine Phalloidin reagent	Rabbit	Ab235138	Abcam	1:100

Table S1b. Secondary antibodies used for immunocytochemistry assays

Secondary antibodies	Host/ Isotype	Catalogue Number	Marker	Company	Working Concentration
Alexa fluor 568	Anti-Rabbit	1704462	Red	Life Technology	1:250
Fluorescein	Anti-Rabbit	1475348	Green	Life Technology	1:250
Cy3	Anti-Mouse	1547506	Red	Life Technology	1:250

Supplementary Methods

Proliferation: WST-1 assay

3 x 10^3 cells were plated in 96-well plates (3596; Corning costar treated plates, Corning, NY, USA) overnight before adding 2 μM PD173074 or DMSO (vehicle control). Following the manufacturer's instructions, the cell proliferation reagent WST-1 (Roche Diagnostics GmbH, Mannheim, Germany) was applied after 24, 48, or 72 h, and the cells were then incubated for 30 to 60 minutes. An infinite plate reader (Tecan, Infinite® 200 pro, Megallan™ software version 7.2 SP1, Männedorf, Switzerland) was then used to measure each sample's absorbance at 420 nm.

Apoptosis: CaspGlow™ Fluorescein active caspase-3

Following the manufacturer's instructions, active caspase-3 was detected using the CaspGlowTM Fluorescein Active caspase-3 staining kit (Thermo Fisher Scientific, Manassas, VA, USA) in order to assess the impact of treatment on apoptosis.
