# Supplementary figures and images for: Development and Characterization of Three Novel FGFR Inhibitor Resistant Cervical Cancer Cell Lines to Help Drive Cervical Cancer Research
Source: Int J Mol Sci. 2025 Feb 20;26(5):1799. doi: 10.3390/ijms26051799 (PMC11898767; doi:10.3390/ijms26051799)

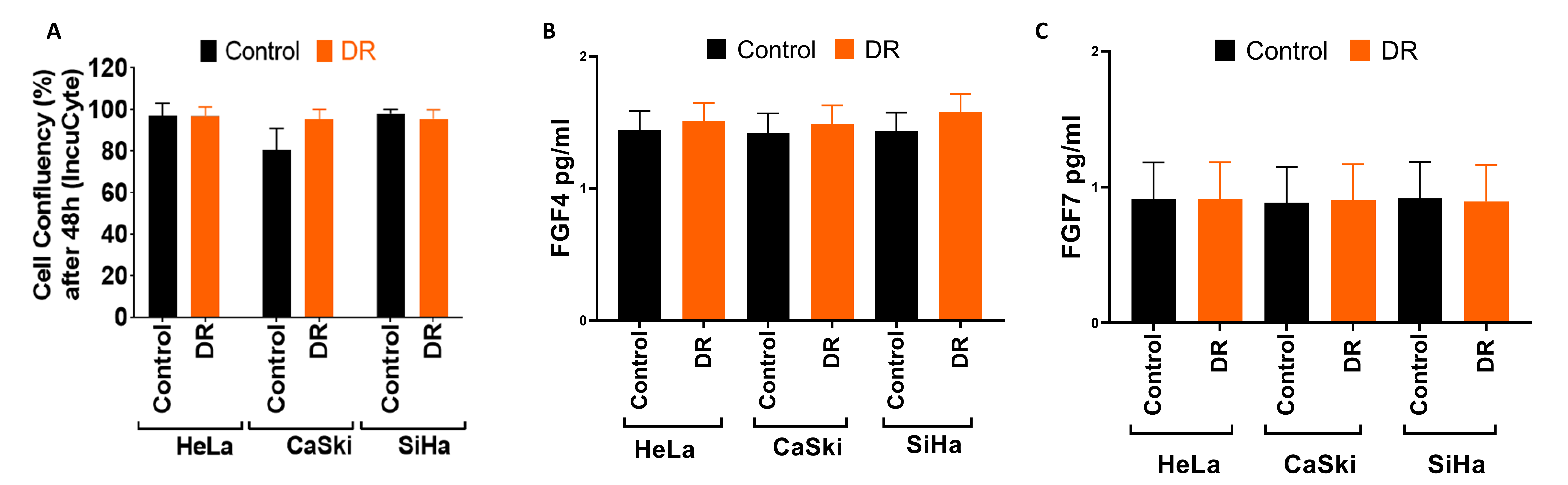

Supplement: Supplementary file 1 [file ijms-26-01799-s001.zip › Figure S1.tif]

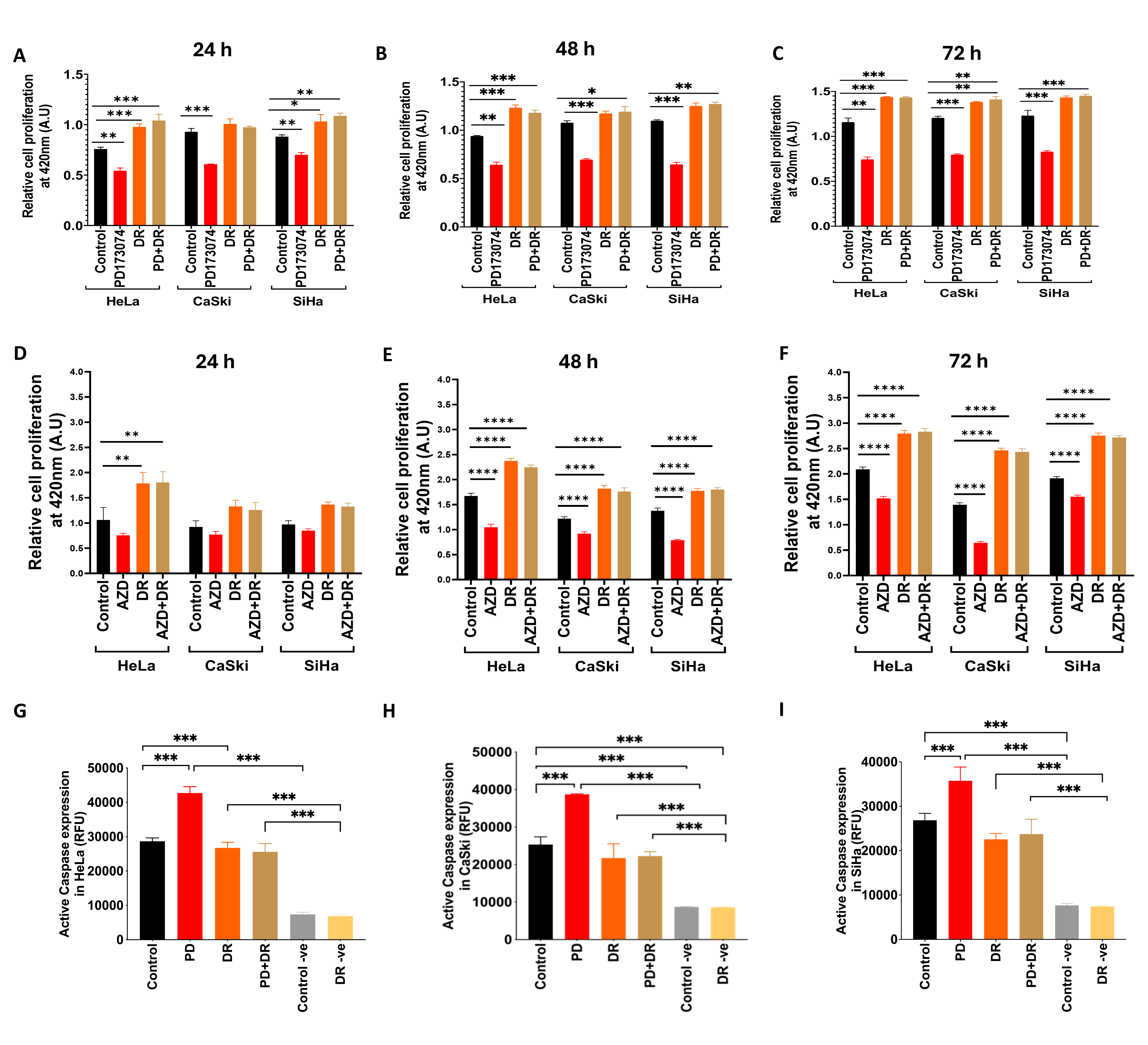

Supplement: Supplementary file 1 [file ijms-26-01799-s001.zip › Figure S2.tif]

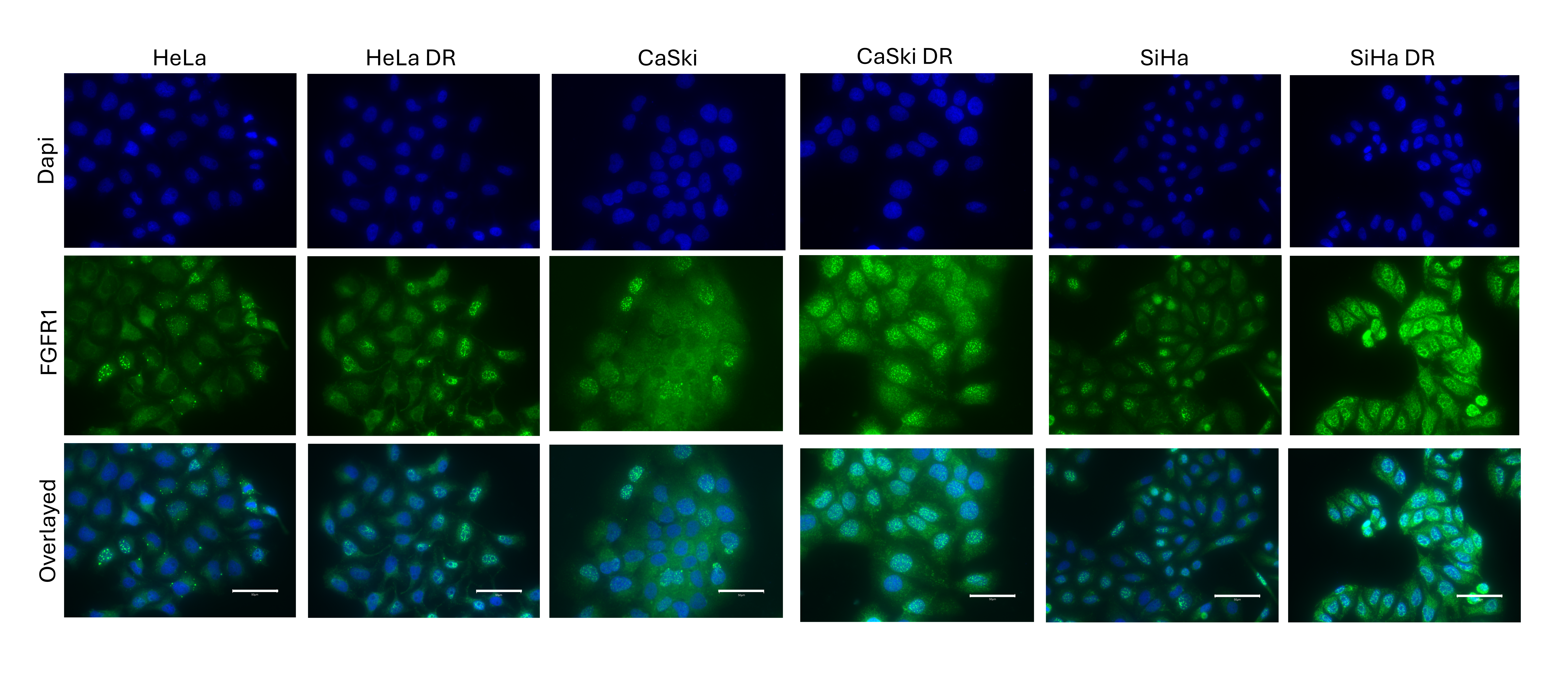

Supplement: Supplementary file 1 [file ijms-26-01799-s001.zip › Figure S3.tif]

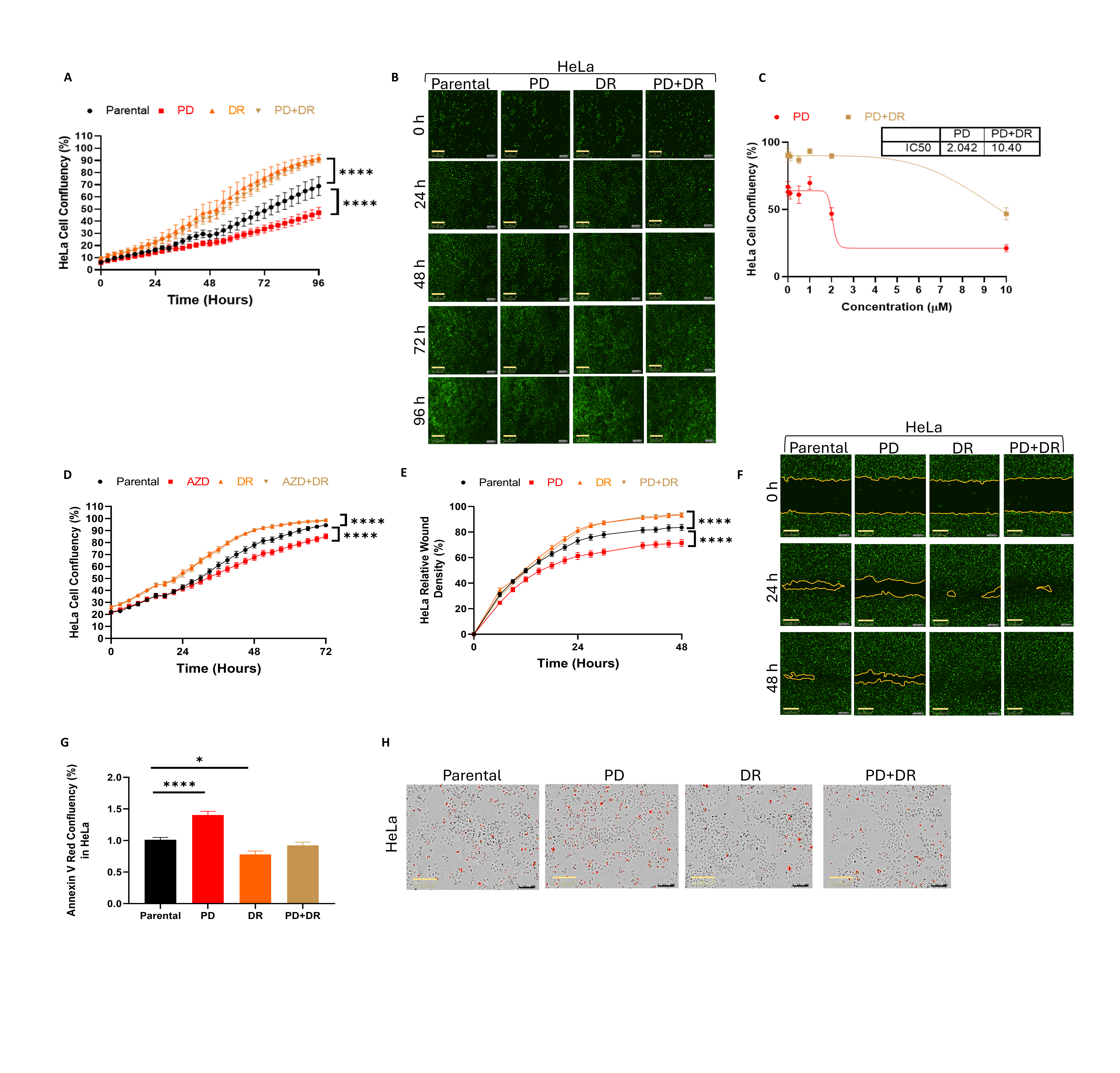

Supplement: Supplementary file 1 [file ijms-26-01799-s001.zip › Figure S4.tif]

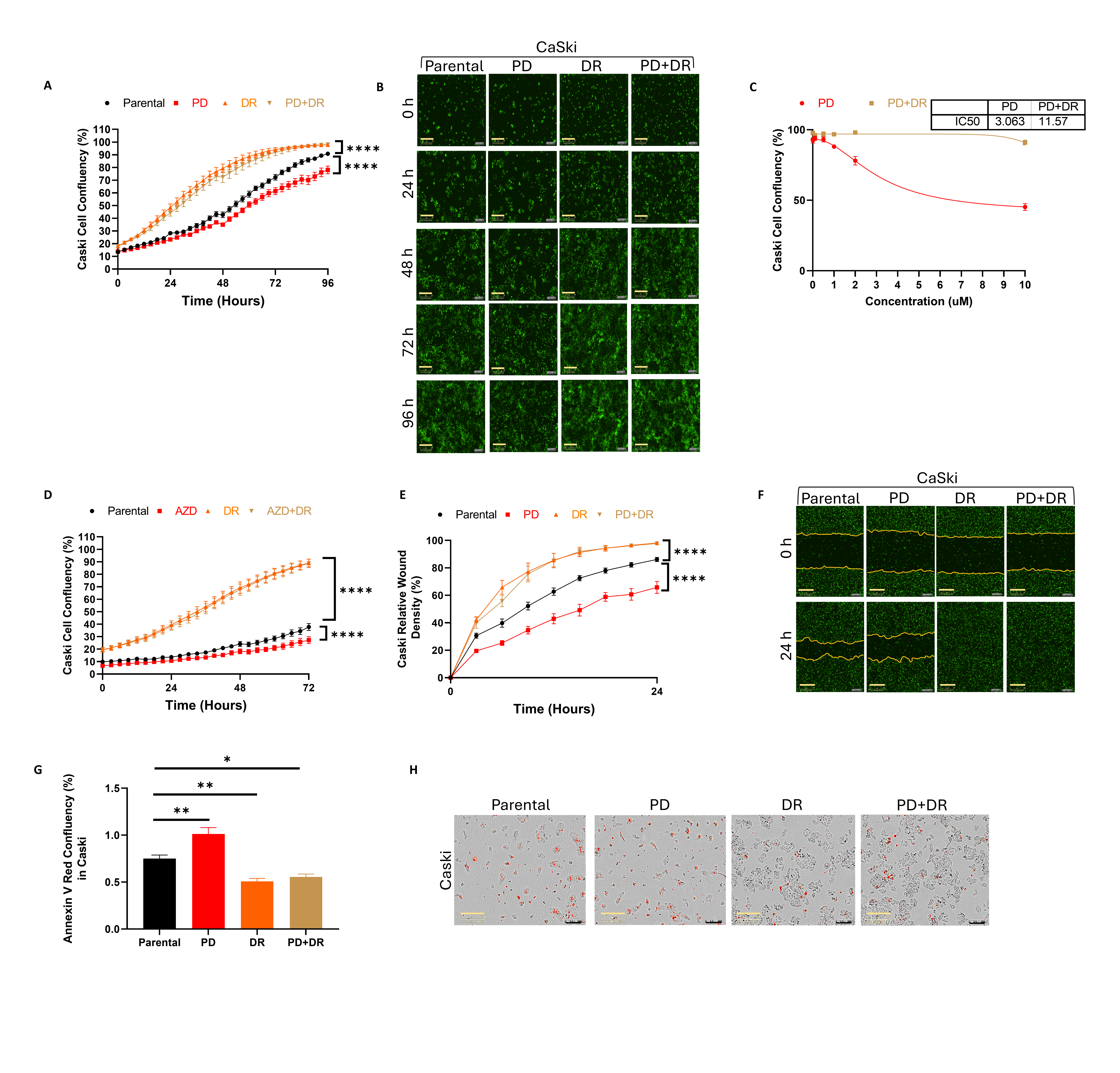

Supplement: Supplementary file 1 [file ijms-26-01799-s001.zip › Figure S5.tif]
